# Supplementary material for: Knowledge, attitude, and practice on blood donation among undergraduate first-year engineering students in Nepal
Source: PLoS One. 2026 May 26;21(5):e0349219. doi: 10.1371/journal.pone.0349219 (PMC13210178; doi:10.1371/journal.pone.0349219)
Supplement: S2 File — (DOCX) [file pone.0349219.s002.docx]

**Questionnaire**

Instructions: Please tick (✓) the appropriate box or write your answer in the provided space. All information will be kept confidential.

**Part I: Socio-Demographic Information**

1. **Age (in years):** _______
2. **Sex:**
   □ Female
   □ Male
3. **Religion:**
   □ Hindu
   □ Buddhist
   □ Christian
   □ Kirat
   □ Muslim
   □ Others (Specify): _________
4. **Ethnicity:**
   □ Brahmin/Chhetri
   □ Janjati
   □ Madhesi
   □ Muslim
   □ Dalit
   □ Others (Specify): _________
5. **You are currently living with:**
   □ Both father and mother
   □ Mother only
   □ Father only
   □ Relatives
   □ Other person
   □ Alone
6. **Academic Programme:**
   □ Civil Engineering (BCE)
   □ Computer Engineering (BCT)
   □ Mechanical Engineering (BME)
   □ Electrical Engineering (BEL)
   □ Electronics, Communication & Information Engineering (BEX)
   □ Aerospace Engineering (BAE)
   □ Chemical Engineering (BCH)
   □ Bachelor of Architecture (BAR)
7. **Father’s highest education level:**
   □ Illiterate
   □ Literate (no formal schooling)
   □ Basic level (up to class 8)
   □ Secondary Level (up to class 12)
   □ University-level
8. **Mother’s highest education level:**
   □ Illiterate
   □ Literate (no formal schooling)
   □ Basic level (up to class 8)
   □ Secondary Level (up to class 12)
   □ University-level
9. **Family residence is located in a:**
   □ Rural Municipality
   □ Municipality
   □ Sub-metropolitan City
   □ Metropolitan City

**Part II: Knowledge**

*For the following statements, please select the single best answer unless specified otherwise.*

1. **The minimum age to start blood donation is:**
   □ 16 years
   □ 18 years
   □ 20 years
   □ Don’t know
2. **The minimum weight required for blood donation is:**
   □ 40 kg
   □ 45 - 50 kg
   □ 60 kg
   □ Don’t know
3. **The minimum hemoglobin level required for blood donation is:**
   □ >12.5 gm/dl
   □ >15 gm/dl
   □ >20.5 gm/dl
   □ Don’t know
4. **The minimum interval between two successive blood donations is:**
   □ 1 month
   □ 3 months
   □ 6 months
   □ Don’t know
5. **The amount of blood that can be donated by a person at a time is:**
   □ 350-470 ml
   □ 500-750 ml
   □ Above 1 liter
   □ Don’t know
6. **The recommended duration to refrain from work after blood donation is:**
   □ Rest not needed
   □ 1 day
   □ 1 week
   □ Don’t know
7. **The time required for the blood level to come back to normal is:**
   □ 1-2 weeks
   □ 6-12 weeks
   □ 15-20 weeks
   □ Don’t know
8. **The kind of food to be taken after blood donation includes:**
   □ Meat, fish, egg, spinach, nuts, pulse, fresh water, etc.
   □ Bread, pizza, cold drinks, burger, etc.
   □ None
   □ Don’t know
9. **The number of patients benefited from 1 unit of whole blood is:**
   □ One patient
   □ Three patients
   □ Ten patients
   □ Don’t know
10. **The most common type of blood group is:**
    □ O+ve
    □ O-ve
    □ None
    □ Don’t know
11. **The minimum duration between delivery of the baby and blood donation is:**
    □ Three months
    □ Nine months
    □ Twelve months
    □ Don’t know
12. **Screening of blood is necessary before donation.**
    □ Yes
    □ No
    □ Don’t know
13. **Infections can be transmitted by blood donation.**
    □ Yes
    □ No
    □ Don’t know
14. **The required blood pressure at the time of blood donation is:**
    □ Systolic BP below 180 and diastolic BP below 100
    □ Systolic BP above 180 and diastolic BP above 100
    □ None
    □ Don’t know
15. **World Blood Donation Day is celebrated on:**
    □ 14th June
    □ 14th August
    □ 14th May
    □ Don’t know
16. **The duration of the blood donation process is:**
    □ About one hour
    □ About two hours
    □ About three hours
    □ Don’t know
17. **The maximum duration platelets can be stored is:**
    □ Five days
    □ Ten days
    □ Thirty days
    □ Don’t know
18. **What are the types of legal blood donors?** (Select all that apply)
    □ Voluntary
    □ Family/Replacement
    □ Paid
    □ All above
    □ Don’t know
19. **Where can one donate blood legally?** (Select all that apply)
    □ Health center
    □ Any company
    □ Community organization
    □ All above
    □ Don’t know

**Part III: Attitude**

1. **Is blood donation a good and noble act?**
   □ Agree
   □ Disagree
   □ Neutral
2. **What is your attitude towards blood donation?**
   □ Positive
   □ Negative
   □ Neutral
3. **What do you think is the best source of blood donors?**
   □ Voluntary
   □ Replacement
   □ Paid
   □ I don’t know
4. **Are you willing to donate blood to relatives?**
   □ Yes
   □ No
5. **Are you willing to donate blood to anyone?**
   □ Yes
   □ No
6. **Will you donate blood without knowing the religion of the recipient?**
   □ Yes
   □ No
7. **Do you expect any reward for blood donation?**
   □ Yes
   □ No

**Part IV: Practice**

1. **Have you donated blood before?**
   □ Yes
   □ No
2. **How many times have you donated blood?**
   □ Once
   □ Twice
   □ Trice
   □ More than three times
3. **Why did you donate blood?**
   □ A relative needed blood
   □ A known person (non-relative) needed blood
   □ Voluntary donation
4. **Are you satisfied after donating blood?**
   □ Yes
   □ No
5. **Are you willing to donate blood in the future?**
   □ Yes
   □ No
6. **What was the reason for not donating blood?** (Select all that apply)
   □ No specific reason
   □ Fear
   □ Parental pressure
   □ Religion
   □ No awareness
   □ No opportunity

□ Other (specify_______)

1. **Have any members of your family donated blood before?**
   □ Yes
   □ No
   □ Don’t know

***Thank you!***
