# Supplementary material for: Knowledge, attitude, and practice on blood donation among undergraduate first-year engineering students in Nepal
Source: PLoS One. 2026 May 26;21(5):e0349219. doi: 10.1371/journal.pone.0349219 (PMC13210178; doi:10.1371/journal.pone.0349219)
Supplement: S3 File — (DOCX) [file pone.0349219.s003.docx]

**Table S1.** Item-Total Statistics for Knowledge Scale (n = 191)

| **Item** | **Scale Mean if Item Deleted** | **Scale Variance if Item Deleted** | **Corrected Item-Total Correlation** | **Cronbach's α if Item Deleted** |
| --- | --- | --- | --- | --- |
| A1 | 5.40 | 0.809 | 0.087 | 0.430 |
| A2 | 5.42 | 0.677 | 0.344 | 0.322 |
| A3 | 5.49 | 0.630 | 0.253 | 0.354 |
| A4 | 5.40 | 0.737 | 0.282 | 0.362 |
| A5 | 5.46 | 0.628 | 0.345 | 0.305 |
| A6 | 5.41 | 0.769 | 0.160 | 0.405 |
| A7 | 5.59 | 0.674 | 0.031 | 0.530 |

Scale statistics: Cronbach's α = 0.43 (7 items); Mean = 6.36, SD = 0.935; Mean corrected item-total correlation = 0.21 (range: 0.03-0.35; 5/7 >0.10); No item deletion substantially improved α (max α = 0.530 without A7)

**Table S2.** EFA Results for Attitude Scale (n = 191)

| **Item** | **Factor 1 Loading** | **Communality** |
| --- | --- | --- |
| A1 | 0.26 | 0.38 |
| A2 | 0.64 | 0.60 |
| A3 | 0.34 | 0.18 |
| A4 | 0.54 | 0.48 |
| A5 | 0.60 | 0.52 |
| A6 | 0.26 | 0.09 |
| A7 | 0.04 | 0.19 |

Model fit statistics: KMO = 0.58 (middling); Bartlett's χ² = 134.81, df = 21, p < 0.001; Factor 1: Eigenvalue = 1.32, 19% variance explained (loadings range: 0.04-0.64); Principal Axis Factoring; unrotated single-factor solution confirms modest one-dimensionality despite ceiling effects and low item correlations.

**Table S3.** Level of knowledge (n=191)

| **Statement (correct answer)** | **N** | **%** |
| --- | --- | --- |
| Minimum age to start blood donation (18 years) | 140 | 73.3 |
| Minimum weight required for blood donation (45 - 50 kg) | 125 | 65.4 |
| Minimum hemoglobin level required for blood donation (>12.5 gm/dl) | 59 | 30.9 |
| The minimum interval between two successive blood donations (3 months). | 127 | 66.5 |
| The amount of blood that can be donated by a person at a time (350-470 ml) | 113 | 59.2 |
| Duration to refrain from doing work after the blood donation (1 day) | 115 | 60.2 |
| Time required for the blood level to come back to normal (6-12 weeks) | 56 | 29.3 |
| Kind of food to be taken after blood donation (Meat, fish, egg, spinach, nuts, pulse, fresh water, etc) | 161 | 84.3 |
| Number of patients benefited from 1 unit of whole blood (Three) | 36 | 18.8 |
| The most common type of blood group (O+ve) | 135 | 70.7 |
| Minimum duration between delivery of the baby and blood donation (9 months) | 34 | 17.8 |
| Screening of blood is necessary before donation (Yes) | 171 | 89.5 |
| Infection transmitted by blood donation (Yes) | 149 | 78.0 |
| Required blood pressure at the time of blood donation (Systolic blood pressure below 180 and diastolic blood pressure below 100) | 95 | 49.7 |
| World Blood Donation Day (14^th^ June) | 77 | 40.3 |
| Duration of the blood donation process (About one hour) | 139 | 72.8 |
| The maximum duration platelets can be stored (Five days) | 56 | 29.3 |
| Types of legal blood donation: * |  |  |
| Voluntary (Yes) | 180 | 94.2 |
| Family replacement (Yes) | 136 | 71.2 |
| Paid (No) | 93 | 48.7 |
| Places of legal blood donation: * |  |  |
| Health center (Yes) | 177 | 92.7 |
| Any Company (No) | 162 | 84.8 |
| Community organization (Yes) | 121 | 63.4 |

*Multiple choice

**Table S4.** Level of Attitude (n=191)

| **Statement (correct answer)** | **N** | **%** |
| --- | --- | --- |
| Is blood donation a good and noble act? (Agree) | 184 | 96.3 |
| What is your attitude towards blood donation? (Positive) | 179 | 97.3 |
| What do you think is the best source of blood donors? (Voluntary) | 167 | 87.4 |
| Are you willing to donate blood to relatives? (Yes) | 183 | 95.8 |
| Are you willing to donate blood to anyone? (Yes) | 173 | 90.6 |
| Will you donate blood without knowing the religion of the recipient? (Yes) | 182 | 95.3 |
| Do you expect any reward for blood donation? (No) | 147 | 77.0 |

**Table S5.** Practice of blood donation

| **Statement** | **Category** | **N** | **%** |
| --- | --- | --- | --- |
| Have you donated blood before? (n=191) | Yes | 32 | 16.8 |
|  | No | 159 | 83.2 |
| How many times have you donated blood? (n=32) | Once | 28 | 87.5 |
|  | Twice | 4 | 12.5 |
| Why did you donate blood? (n=32) | A relative needed blood | 2 | 6.3 |
|  | Voluntary | 30 | 93.7 |
| Are you satisfied after donating blood? (n=32) | Yes | 31 | 96.9 |
|  | No | 1 | 3.1 |
| Are you willing to donate blood in the future? (n=32) | Yes | 30 | 93.8 |
|  | No | 2 | 6.2 |
| What was the reason for not donating blood?* (n=159) | No specific reason | 68 | 42.8 |
|  | Fear | 32 | 20.1 |
|  | Parental pressure | 5 | 3.1 |
|  | No awareness | 9 | 5.7 |
|  | No opportunity | 55 | 34.6 |
|  | Ineligible | 16 | 10.1 |
|  | Medical condition | 9 | 5.7 |
| Have any members of your family donated blood before? (n=191) | Yes | 130 | 68.1 |
|  | No | 61 | 31.9 |

*Multiple choice question

**Table S6.** Knowledge Scores by Sociodemographic Characteristics (n=191)

| **Variable** | **Category** | **N** | **Mean (SD)** | **t** | **df** | **p-value** | **Mean Diff (95% CI)** | **Cohen's d** |
| --- | --- | --- | --- | --- | --- | --- | --- | --- |
| Age | <20 years | 87 | 14.16 (4.02) | 0.83 | 189 | 0.408 | 0.46  (-0.63 to 1.55) | 0.12 |
|  | ≥20 years | 104 | 13.70 (3.63) |  |  |  |  |  |
| Sex | Female | 51 | 14.96 (3.69) | 2.33 | 189 | 0.021* | 1.43  (0.22 to 2.65) | 0.38 |
|  | Male | 140 | 13.53 (3.79) |  |  |  |  |  |
| Religion | Non-Hindu^a^ | 13 | 14.15 (3.31) | 0.24 | 189 | 0.812 | 0.26  (-1.90 to 2.42) | 0.07 |
|  | Hindu | 178 | 13.89 (3.85) |  |  |  |  |  |
| Ethnicity | Non-Brahmin/Chhetri^b^ | 53 | 14.51 (3.46) | 1.35 | 189 | 0.179 | 0.83  (-0.38 to 2.04) | 0.22 |
|  | Brahmin/Chhetri | 138 | 13.68 (3.92) |  |  |  |  |  |
| Living with | Non-nuclear^c^ | 61 | 13.66 (4.26) | -0.63 | 189 | 0.527 | -0.38  (-1.54 to 0.79) | -0.10 |
|  | Both parents | 130 | 14.03 (3.59) |  |  |  |  |  |
| Program | Other engineering^d^ | 96 | 14.27 (3.68) | 1.32 | 189 | 0.190 | 0.72  (-0.36 to 1.81) | 0.19 |
|  | Civil engineering | 95 | 13.55 (3.92) |  |  |  |  |  |
| Father's education | Basic or below | 45 | 13.82 (3.90) | -0.18 | 189 | 0.859 | -0.12  (-1.40 to 1.17) | -0.03 |
|  | Secondary or above | 146 | 13.94 (3.79) |  |  |  |  |  |
| Mother's education | Basic or below | 77 | 13.88 (4.32) | -0.08 | 189 | 0.934 | -0.05  (-1.16 to 1.06) | -0.01 |
|  | Secondary or above | 114 | 13.93 (3.44) |  |  |  |  |  |
| Residency | Municipality | 109 | 14.14 (4.23) | 0.95 | 189 | 0.344 | 0.53  (-0.57 to 1.63) | 0.14 |
|  | Metropolitan city | 82 | 13.61 (3.16) |  |  |  |  |  |

Non-Hindu^a^ includes Buddhist, Christian, Kirat, and Muslim; Non-Brahmin/Chhetri^b^ includes Janjati, Madhesi, Muslim, and Dalit; Non-nuclear^c^ includes living alone, with only father, only mother, and other people; Other engineering^d^ includes mechanical engineering, Electronics, Communication, & Information Engineering, Computer Engineering, Electrical Engineering, Aerospace Engineering, Chemical Engineering, and Bachelor of Architecture; *p < .05

**Table S7**. Attitude Scores by Sociodemographic Groups (n = 191)

| **Characteristic** | **Category** | **N** | **Mean Rank** | **U** | **Z** | **p** |
| --- | --- | --- | --- | --- | --- | --- |
| Age | <20 years | 87 | 97.7 | 4373 | -0.45 | 0.653 |
|  | ≥20 years | 104 | 94.5 |  |  |  |
| Sex | Female | 51 | 101.1 | 3310 | -0.87 | 0.386 |
|  | Male | 140 | 94.1 |  |  |  |
| Religion | Non-Hindu^a^ | 13 | 68.3 | 797 | -2.11 | 0.035* |
|  | Hindu | 178 | 98.0 |  |  |  |
| Ethnicity | Non-Brahmin/Chhetri^b^ | 53 | 90.5 | 3365 | -0.97 | 0.335 |
|  | Brahmin/Chhetri | 138 | 98.1 |  |  |  |
| Living with | Non-nuclear^c^ | 61 | 80.9 | 3047 | -2.91 | 0.004* |
|  | Both parents | 130 | 103.1 |  |  |  |
| Programme | Other engineering^d^ | 96 | 98.0 | 4365 | -0.58 | 0.565 |
|  | Civil engineering | 95 | 93.9 |  |  |  |
| Father’s education | Basic or below | 45 | 93.8 | 3187 | -0.34 | 0.733 |
|  | Secondary or above | 146 | 96.7 |  |  |  |
| Mother’s education | Basic or below | 77 | 86.9 | 3686 | -2.12 | 0.034* |
|  | Secondary or above | 114 | 102.2 |  |  |  |
| Residency | Municipality | 109 | 91.5 | 3981 | -1.46 | 0.145 |
|  | Metropolitan City | 82 | 102.0 |  |  |  |

Non-Hindu^a^ includes Buddhist, Christian, Kirat, and Muslim; Non-Brahmin/Chhetri^b^ includes Janjati, Madhesi, Muslim, and Dalit; Non-nuclear^c^ includes living alone, with only father, only mother, and other people; Other engineering^d^ includes mechanical engineering, Electronics, Communication, & Information Engineering, Computer Engineering, Electrical Engineering, Aerospace Engineering, Chemical Engineering, and Bachelor of Architecture; *p < .05

**Table S8.** Spearman’s Rho Correlation Matrix of Knowledge scores, Attitude scores, and Practice

| **Variables** | **Knowledge Score** | **Attitude Score** | **Practice** |
| --- | --- | --- | --- |
| Knowledge Score | 1.000 |  |  |
| Attitude Score | 0.176* (p=0.015) | 1.000 |  |
| Practice | 0.053 (p=0.466) | 0.045 (p=0.532) | 1.000 |

*p < .05

**Table S9.** Blood Donation Practice by Sociodemographic Characteristics (n = 191)

| **Characteristic** | **Category** | **No (N, %)** | **Yes (N, %)** | **χ² (df = 1)** | **p-value** |
| --- | --- | --- | --- | --- | --- |
| Age | <20 years | 80 (92.0) | 7 (8.0) | 8.687 | 0.003* |
|  | ≥20 years | 79 (76.0) | 25 (24.0) |  |  |
| Sex | Female | 48 (94.1) | 3 (5.9) | 5.896 | 0.015* |
|  | Male | 111 (79.3) | 29 (20.7) |  |  |
| Religion | Non-Hindu | 10 (76.9) | 3 (23.1) | 0.400 | 0.527 |
|  | Hindu | 149 (83.7) | 29 (16.3) |  |  |
| Ethnicity | Non-Brahmin/Chhetri | 43 (81.1) | 10 (18.9) | 0.235 | 0.628 |
|  | Brahmin/Chhetri | 116 (84.1) | 22 (15.9) |  |  |
| Living with | Non-nuclear | 51 (83.6) | 10 (16.4) | 0.008 | 0.927 |
|  | Both parents | 108 (83.1) | 22 (16.9) |  |  |
| Programme | Other engineering | 75 (78.1) | 21 (21.9) | 3.629 | 0.057 |
|  | Civil engineering | 84 (88.4) | 11 (11.6) |  |  |
| Father’s education | Basic or below | 34 (75.6) | 11 (24.4) | 2.496 | 0.114 |
|  | Secondary or above | 125 (85.6) | 21 (14.4) |  |  |
| Mother’s education | Basic or below | 61 (79.2) | 16 (20.8) | 1.499 | 0.221 |
|  | Secondary or above | 98 (86.0) | 16 (14.0) |  |  |
| Residency | Municipality | 90 (82.6) | 19 (17.4) | 0.083 | 0.773 |
|  | Metropolitan City | 69 (84.1) | 13 (15.9) |  |  |

Non-Hindu^a^ includes Buddhist, Christian, Kirat, and Muslim; Non-Brahmin/Chhetri^b^ includes Janjati, Madhesi, Muslim, and Dalit; Non-nuclear^c^ includes living alone, with only father, only mother, and other people; Other engineering^d^ includes mechanical engineering, Electronics, Communication, & Information Engineering, Computer Engineering, Electrical Engineering, Aerospace Engineering, Chemical Engineering, and Bachelor of Architecture; *p < .05

**Table S10.** Association Between Socio-demographics and Binary Knowledge (≥50th Percentile Adequate; n=191)

| **Variable** | **Category** | **Inadequate (N, %)** | **Adequate (N, %)** | **χ² (df=1)** | **p-value** |
| --- | --- | --- | --- | --- | --- |
| Age | <20 years | 36 (41.4) | 51 (58.6) | 2.51 | 0.113 |
|  | ≥20 years | 55 (52.9) | 49 (47.1) |  |  |
| Sex | Female | 19 (37.3) | 32 (62.7) | 3.01 | 0.083 |
|  | Male | 72 (51.4) | 68 (48.6) |  |  |
| Religion | Non-Hindu^a^ | 7 (53.8) | 6 (46.2) | 0.22 | 0.643 |
|  | Hindu | 84 (47.2) | 94 (52.8) |  |  |
| Ethnicity | Non-Brahmin/Chhetri^b^ | 23 (43.4) | 30 (56.6) | 0.53 | 0.466 |
|  | Brahmin/Chhetri | 68 (49.3) | 70 (50.7) |  |  |
| Living with | Non-nuclear^c^ | 30 (49.2) | 31 (50.8) | 0.09 | 0.771 |
|  | Both parents | 61 (46.9) | 69 (53.1) |  |  |
| Program | Other engineering^d^ | 41 (42.7) | 55 (57.3) | 1.89 | 0.170 |
|  | Civil engineering | 50 (52.6) | 45 (47.4) |  |  |
| Father's education | Basic or below | 23 (51.1) | 22 (48.9) | 0.28 | 0.594 |
|  | Secondary or above | 68 (46.6) | 78 (53.4) |  |  |
| Mother's education | Basic or below | 40 (51.9) | 37 (48.1) | 0.96 | 0.328 |
|  | Secondary or above | 51 (44.7) | 63 (55.3) |  |  |
| Residency | Municipality | 51 (46.8) | 58 (53.2) | 0.07 | 0.785 |
|  | Metropolitan City | 40 (48.8) | 42 (51.2) |  |  |

Non-Hindu^a^ includes Buddhist, Christian, Kirat, and Muslim; Non-Brahmin/Chhetri^b^ includes Janjati, Madhesi, Muslim, and Dalit; Non-nuclear^c^ includes living alone, with only father, only mother, and other people; Other engineering^d^ includes mechanical engineering, Electronics, Communication, & Information Engineering, Computer Engineering, Electrical Engineering, Aerospace Engineering, Chemical Engineering, and Bachelor of Architecture

**Table S11.** Binary Logistic Regression Predicting Adequate Knowledge (≥50th Percentile; n=191)

| **Predictor** | **B** | **SE** | **Wald** | **p-value** | **OR** | **95% CI Lower** | **95% CI Upper** |
| --- | --- | --- | --- | --- | --- | --- | --- |
| Age ≥ 20 years (ref: <20 years) | -0.40 | 0.30 | 1.70 | 0.193 | 0.67 | 0.37 | 1.22 |
| Sex Male (ref: Female) | -0.40 | 0.35 | 1.27 | 0.260 | 0.67 | 0.34 | 1.34 |
| Program Civil engineering (ref: Other engineering) | -0.34 | 0.30 | 1.24 | 0.266 | 0.72 | 0.40 | 1.29 |

Model: χ²=5.78 (df=3), p=.123; Nagelkerke R²=0.04; Hosmer-Lemeshow p=.628 (good fit); Other engineering includes mechanical engineering, Electronics, Communication, & Information Engineering, Computer Engineering, Electrical Engineering, Aerospace Engineering, Chemical Engineering, and Bachelor of Architecture

**Table S12.** Association Between Sociodemographic Characteristics and Positive Attitude Score (≥50th Percentile) (n = 191)

| **Characteristic** | **Category** | **Negative (N, %)** | **Positive (N, %)** | **χ² (df = 1)** | **p-value** |
| --- | --- | --- | --- | --- | --- |
| Age | <20 years | 36 (41.4) | 51 (58.6) | 0.157 | 0.692 |
|  | ≥20 years | 46 (44.2) | 58 (55.8) |  |  |
| Sex | Female | 20 (39.2) | 31 (60.8) | 0.392 | 0.531 |
|  | Male | 62 (44.3) | 78 (55.7) |  |  |
| Religion | Non-Hindu^a^ | 9 (69.2) | 4 (30.8) | 3.938 | 0.047* |
|  | Hindu | 73 (41.0) | 105 (59.0) |  |  |
| Ethnicity | Non-Brahmin/Chhetri^b^  Brahmin/Chhetri | 25 (47.2) | 28 (52.8) | 0.538 | 0.463 |
|  |  | 57 (41.3) | 81 (58.7) |  |  |
| Living with | Non-nuclear^c^ | 36 (59.0) | 25 (41.0) | 9.464 | 0.002* |
|  | Both parents | 46 (35.4) | 84 (64.6) |  |  |
| Program | Other engineering^d^ | 39 (40.6) | 57 (59.4) | 0.419 | 0.517 |
|  | Civil engineering | 43 (45.3) | 52 (54.7) |  |  |
| Father's education | Basic or below | 20 (44.4) | 25 (55.6) | 0.055 | 0.815 |
|  | Secondary or above | 62 (42.5) | 84 (57.5) |  |  |
| Mother's education | Basic or below | 40 (51.9) | 37 (48.1) | 4.280 | 0.039* |
|  | Secondary or above | 42 (36.8) | 72 (63.2) |  |  |
| Residency | Municipality | 52 (47.7) | 57 (52.3) | 2.362 | 0.124 |
|  | Metropolitan City | 30 (36.6) | 52 (63.4) |  |  |

Non-Hindu^a^ includes Buddhist, Christian, Kirat, and Muslim; Non-Brahmin/Chhetri^b^ includes Janjati, Madhesi, Muslim, and Dalit; Non-nuclear^c^ includes living alone, with only father, only mother, and other people; Other engineering^d^ includes mechanical engineering, Electronics, Communication, & Information Engineering, Computer Engineering, Electrical Engineering, Aerospace Engineering, Chemical Engineering, and Bachelor of Architecture; *p < .05

**Table S13.** Spearman Correlation Between Knowledge Score and Attitude Category (n=191)

| **Variables** | **Spearman’s rho** | **p-value** |
| --- | --- | --- |
| Knowledge total score vs. Attitude high (≥50th percentile) | 0.153 | 0.034* |

*p < .05

**Table S14.** Binary Logistic Regression Predicting High Attitude (≥50th Percentile; n=191)

| **Predictor** | **B** | **SE** | **Wald** | **p-value** | **OR** | **95% CI Lower** | **95% CI Upper** |
| --- | --- | --- | --- | --- | --- | --- | --- |
| Hindu (ref: Non-Hindu) | 1.20 | 0.65 | 3.44 | 0.064 | 3.31 | 0.94 | 11.69 |
| Living with both parents (ref: Non-nuclear) | 0.88 | 0.34 | 6.81 | 0.009* | 2.40 | 1.24 | 4.63 |
| Mother's education Secondary (ref: Basic or below) | 0.34 | 0.33 | 1.03 | 0.311 | 1.40 | 0.73 | 2.69 |
| Metropolitan City (ref: Municipality) | 0.24 | 0.33 | 0.51 | 0.476 | 1.27 | 0.66 | 2.43 |
| Knowledge score | 0.09 | 0.04 | 4.41 | 0.036* | 1.09 | 1.01 | 1.18 |

Model fit: χ²=20.07 (df=5, p=.001), Nagelkerke R²=13.4%, Hosmer-Lemeshow p=.685; Non-Hindu includes Buddhist, Christian, Kirat, and Muslim; Non-nuclear includes living alone, with only father, only mother, and other people; *p < .05

**Table S15.** Sensitivity Analysis: Reduced Logistic Regression Model Predicting Blood Donation Practice (n = 191)

| Predictor | B | SE | Wald | p | OR | 95% CI Lower | 95% CI Upper |
| --- | --- | --- | --- | --- | --- | --- | --- |
| Age ≥20 years (ref: <20 years) | 1.078 | 0.470 | 5.254 | 0.022* | 2.94 | 1.17 | 7.38 |
| Male (ref: female) | 1.457 | 0.656 | 4.928 | 0.026* | 4.29 | 1.19 | 15.54 |
| Civil eng. (ref: Other engineering) | -0.996 | 0.426 | 5.472 | 0.019* | 0.37 | 0.16 | 0.85 |

Model fit statistics: Hosmer-Lemeshow χ² = 6.31, df = 6, p = 0.390; Nagelkerke R² = 0.161; Omnibus χ² = 19.26, df = 3, p < 0.001; EPV = 10.7 (32 events/3 predictors); Reduced model excludes father education (p = 0.663 in main model). Reference categories consistent with Table 1; *p < 0.05
