# Supplementary material for: Knowledge, attitude, and practice on blood donation among undergraduate first-year engineering students in Nepal
Source: PLoS One. 2026 May 26;21(5):e0349219. doi: 10.1371/journal.pone.0349219 (PMC13210178; doi:10.1371/journal.pone.0349219)
Supplement: Attachment — Submitted filename: Response to Reviewers.docx [file pone.0349219.s006.docx]

Bhola Teli, MPH

B.P. Koirala Institute of Health Science (BPKIHS), Dharan, Nepal

[bholateli23@gmail.com](mailto:bholateli23@gmail.com)

April 13, 2026

Nasar Alwahaibi, PhD

Academic Editor

PLOS ONE

Re: Major Revision - PONE-D-26-05598

Dear Dr. Alwahaibi,

Thank you for the opportunity to revise our manuscript. We appreciate the valuable feedback from Reviewers Temesgen Anjulo Ageru and Beesan Maraqa.

Summary of Revisions:

Journal Requirements:

1. PLOS ONE formatting confirmed using templates

2. Funding text removed (page 32)

3. Figures extracted to separate TIFF files

4. S4 fully anonymized (Age column removed)

5. No mandatory citations

Reviewer 1 - Major Comment 1: Response Rate

No demographic comparison possible (email-only access). Female over-representation (26.7% vs national ~17%) acknowledged (page 30). Post-hoc power 91% (page 14).

Reviewer 1 - Major Comment 2: Logistic Regression

EPV=8 acceptable (Vittinghoff 2007); sensitivity analysis confirms robustness (S13 Table, page 24); Hosmer-Lemeshow p=0.061 (page 23).

Reviewer 1 - Major Comment 3: Attitude Scale α=0.43

Item-total correlations (mean r=0.21, S1 Table); EFA confirms one-dimensionality (KMO=0.58, S2 Table, page 14).

Reviewer 2 - Comments 1-6: Cross-referenced to Reviewer 1 responses and Comment 5 cultural factors added.

We believe these revisions fully address all concerns.

Sincerely,

Bhola Teli, MPH

B.P. Koirala Institute of Health Science (BPKIHS), Dharan, Nepal

[bholateli23@gmail.com](mailto:bholateli23@gmail.com)

Corresponding Author

**Journal Requirements**

**1. PLOS ONE Style Requirements**

**Response:** Manuscript reformatted using PLOS ONE templates (title/authors/affiliations and main body). File naming standardized per guidelines.

**Manuscript changes:** Applied PLOS ONE formatting throughout (tracked changes); files labeled: “Response to Reviewers”, “Manuscript”, “Revised Manuscript with Track Changes”.

**2. Funding Statement**

**Response:** All funding-related text removed from Acknowledgments and manuscript body. Funding Statement unchanged: "The author(s) received no specific funding for this work." No updates required to the online submission form.

**Manuscript changes:** Funding text deleted from Acknowledgments section (page 32, lines 605-607, tracked changes).

**3. Figures**

**Response:** Figure is removed from the manuscript body. Uploaded separately as an individual TIFF file (Fig 1) at 300 dpi per PLOS ONE guidelines. The figure legend is retained at the end of the respective paragraph.

**4. Identifying Data in S4 Minimal Dataset**

**Response:** Ethics committee (Nepal Health Research Council IRB) confirmed participant consent permits fully anonymized data sharing. Removed "<Age>" column (exact ages are direct identifiers). All other variables are coded categorically (0/1 or grouped) with no names, IDs, dates, locations, or contact information. Re-uploaded as "S4 File v2". Dataset now contains only: sex, religion, ethnicity, living_S, Pregramme, father_edu, mother_edu, family_resid (all 0/1 coded). No hidden columns, no indirect identifiers in combination.

**5. Citation of Previously Published Works**

**Response:** No specific citation recommendations provided by reviewers.

**Reviewer #1**

**Major Comment 1: Response Rate and Potential Selection Bias**

**Query 1: Were demographic characteristics of respondents compared with the full eligible population?**

**Response:** No, demographic characteristics of respondents were not compared with the full eligible population, as institutional access provided only email IDs without linked demographic data. However, our sample shows female over-representation (26.7%) compared to national engineering enrollment (~17% female), indicating selection bias toward more responsive female students and limiting gender generalizability. This is now explicitly addressed.

**Manuscript changes:** Non-respondent demographics are unavailable (email-only access), limiting population-sample discrepancy exploration. Female over-representation (26.7% vs. national ~17%) suggests response-propensity bias rather than population composition; it limits gender generalizability (page 30, lines 564-567, tracked changes).

**Query 2: Was any assessment of non-response bias conducted?**

**Response:** No formal assessment of non-response bias was conducted, as we lacked individual-level data for non-respondents beyond email IDs. However, the census-like invitation to all 576 eligible students via a single email platform minimized differential access bias. We hypothesize response bias toward more motivated/accessible students (over-representing females 26.7% vs. national ~17%), potentially inflating positive attitudes/practices.

**Query 3: How does the lower-than-planned sample size affect statistical power and model stability?**

**Response:** Achieved n=191 exceeds unadjusted target n=176. Post-hoc G*Power confirms logistic regression 91% power (OR=2.0, n=191, α=0.05) despite EPV=8, with good model fit (Hosmer-Lemeshow p=0.061).

**Manuscript changes:** Achieved sample size (n=191) exceeds unadjusted target (n=176) (page 10, line 183, tracked change). Post-hoc power analysis of the multivariable logistic regression (blood donation practice; EPV=8) yielded 91% power (GPower; OR=2.0, n=191, α=0.05) (page 14, lines 259-260, tracked changes). The multivariable logistic regression showed acceptable fit (Hosmer-Lemeshow χ²=14.92, df=8, p=0.061), explained 16.3% of variance (Nagelkerke R²=0.163), and maintained validity at EPV=8 (acceptable per Vittinghoff & McCulloch, 2007) (page 23, lines 404-407, tracked changes).

**Major Comment 2: Sample Size and Logistic Regression Stability**

**Query 1: Was EPV formally assessed?**

**Response:** Yes, EPV=8 (32 events/4 predictors). While ≥10 is conservative, EPV=8 yields acceptable bias for hypothesis testing per Vittinghoff & McCulloch (2007).

**Manuscript changes:** The multivariable logistic regression showed acceptable fit (Hosmer-Lemeshow χ²=14.92, df=8, p=0.061), explained 16.3% of variance (Nagelkerke R²=0.163), and maintained validity at EPV=8 (acceptable per Vittinghoff & McCulloch, 2007) (page 23, lines 404-407, tracked changes).

**Query 2: Were alternative modelling strategies considered (e.g., reduced models)?**

**Response:** Yes, sensitivity analysis tested a reduced model excluding father education (n=3 predictors, EPV=10.7). Results confirmed main model robustness: similar Nagelkerke R² (0.163→0.161) and superior fit (p=0.061→0.390).

**Manuscript changes:** Given the borderline EPV=8, sensitivity analysis tested a reduced model excluding father education (p = 0.663 in the main model) (page 14, lines 260-262, tracked changes). For the practice of blood donation, the reduced model (age, sex, program; EPV=10.7) confirmed the robustness of the main findings: superior fit (Hosmer-Lemeshow χ²=6.31, df=6, p=0.390) and equivalent explanatory power (Nagelkerke R²=0.161 vs 0.163) as shown in S13 Table (pages 24-25, lines 438-441, tracked changes).

**Query 3: Was model goodness-of-fit evaluated (e.g., Hosmer–Lemeshow test)?**

**Response:** Yes, model goodness-of-fit is evaluated.

**Manuscript changes:** The multivariable logistic regression showed acceptable fit (Hosmer-Lemeshow χ²=14.92, df=8, p=0.061), explained 16.3% of variance (Nagelkerke R²=0.163), and maintained validity at EPV=8 (acceptable per Vittinghoff & McCulloch, 2007) (page 23, lines 404-407, tracked changes).

**Major Comment 3: Internal Consistency of Attitude Scale (Cronbach's α=0.43)**

**Query 1: Were item-total correlations assessed?**

**Response:** Yes, item-total correlations formally assessed (mean r = 0.21, range 0.03-0.35; 5/7 >0.10 threshold). Maximum α = 0.53 vs original 0.43 (S1 Table).

**Manuscript changes:** Internal consistency of both questionnaires was assessed using Cronbach's α. The knowledge scale showed acceptable reliability (α = 0.73), while the attitude scale had lower but reported reliability (α = 0.43; mean corrected item-total r = 0.21 ± 0.09, range: 0.03-0.35; S1 Table) (page 14, lines 262-265, tracked changes).

**Query 2: Was exploratory factor analysis considered?**

**Response:** Yes, EFA conducted (KMO=0.58 middling; Bartlett's p<0.001; eigenvalue=1.32, 19% variance, loadings 0.04-0.64). Confirms modest one-dimensionality despite ceiling effects

**Manuscript changes:** EFA confirmed modest attitude scale one-dimensionality (KMO=0.58; Bartlett's p<0.001; 1 factor, eigenvalue=1.32 explaining 19% variance; loadings 0.04-0.64), as shown in the S2 Table (page 14, lines 265-267, tracked changes).

**Query 3: How do authors justify using this scale in multivariable linear regression?**

**Response:** Attitude scale justified by EFA confirming one-dimensionality (KMO=0.58; 1 factor; S2 Table). Low α=0.43 was already acknowledged in the Limitations section; findings interpreted as exploratory.

**Major Comment 4: Interpretation of Regression Models**

**4.1 Knowledge Model (F=2.52, p=0.060, R²=0.04)**

**Response:** Despite a borderline non-significant overall model (F=2.52, p=0.060, R²=0.04), the individual sex effect remains statistically reliable (B=-1.26, 95% CI: -2.51 to -0.01, p=0.048). Standard statistical practice allows interpretation of significant individual predictors regardless of the overall model F-test or R² magnitude. Sex finding is valid and interpretable.

**4.2 Attitude Model (R²=0.07, B=0.04)**

**Response:** Practical significance now appropriately cautious: B=0.04 indicates a statistically significant but trivial effect (0.04-point attitude change per knowledge point).

**Manuscript changes:** Knowledge score was the sole significant predictor of attitude (B = 0.04, p = 0.025), indicating a statistically significant but very small association. A one-unit knowledge increase predicts only a 0.04-point attitude improvement, suggesting minimal practical impact (page 28, lines 511-513, tracked changes).

**4.3 Knowledge–Practice Gap**

**Query 1: How operationally defined?**

**Response:** Descriptive discrepancy between high knowledge/attitude scores (≥50th percentile = "adequate/positive") and low practice prevalence (17% ever donated, n=32/191).

**Query 2: Descriptive vs. analytic?**

**Response:** Descriptive, not analytic. "Knowledge-practice gap" refers purely to the observed pattern of favorable K/A scores alongside low donation rates, not mediation or causation.

**Manuscript changes:** A descriptive "knowledge-practice gap" exists among engineering students. While females are more knowledgeable, males are more likely to donate (page 3, lines 33-34, tracked changes). This descriptive "knowledge-practice gap" suggests that while students are ideologically willing to donate, structural or personal barriers—rather than a lack of awareness—may be hindering actual donation (page 25, lines 450-452, tracked changes). Our findings highlight a descriptive "knowledge-practice gap," where behavior is primarily driven by age, sex, and academic program rather than awareness alone (page 31, lines 586-588, tracked change).

**Major Comment 5: Causal Language in Discussion**

**Response:** All causal language has been systematically revised throughout the manuscript (tracked changes). Cross-sectional limitation is already explicitly stated in the Limitations section that the study design does not establish causal relationships.

**Major Comment 6: Dichotomization of Variables**

**Query 1: Why age was not treated continuously?**

**Response:** Age categorized at median (20 years) following a data-driven approach and international precedent [Eltewacy et al. 2024 using identical <20/≥20 cutoff]. Captures meaningful threshold effect distinguishing late adolescents from young adults, avoiding the linearity assumption unsupported by behavioral data. [Royston & Altman, 2006]

**Manuscript changes:** This was treated as a categorical variable, dichotomized into "Less than 20 years" and "20 years or more" based on the sample distribution, previous study, and avoiding linearity assumption unsupported by behavioral data (page 12, lines 213-215, tracked changes).

**Query 2: Why median splits rather than evidence-based thresholds?**

**Response:** Median splits are used only in sensitivity analyses as a data-driven approach, ensuring balanced groups, standard practice when no established clinical thresholds exist for student knowledge/attitude scores.

**Major Comment 7: Social Desirability and Ceiling Effect**

**Response:** S2 Table clearly shows the ceiling effect (90-97% positive responses across 5/7 items, median=7/7), which naturally explains the low α=0.43 due to limited variability and social desirability bias common in Nepal donation surveys. This also accounts for the small regression coefficient (B=0.04); the issue is fully addressed through Comment 3 psychometrics (EFA S2 Table, item-total S1 Table) and in the limitations.

**Minor Comments 1-6**

**1. Physiological description of blood in the Introduction**

**Response:** We have condensed the introduction section, including a physiological description, in the tracked changes.

**2. More recent WHO data beyond 2018**

**Response:** To our knowledge, no comprehensive WHO Global Database on Blood Safety (GDBS) report beyond the 2018 data has been published.

**3. Male proportion (73.3%) vs. campus enrollment**

**Response:** This is already addressed in Major Comment 1, where we explained that demographic data for the full eligible population was unavailable (email IDs only, no linked demographics). This data limitation prevents direct comparison of our 73.3% male proportion against campus-specific enrollment distribution.

**4. Speculative explanations labelled as hypotheses**

**Response:** This has been addressed in tracked changes. Speculative explanations (e.g., academic workload differences between programs) are now clearly labelled as hypotheses in the revised manuscript.

**5. Terminology consistency**

**Response:** We have double-checked the manuscript and ensured consistent use of "knowledge-practice gap" throughout.

**6. Report logistic model fit statistics**

**Response:** This is already addressed in Major Comment 2 (Query 3). Model fit statistics for binary logistic regression on blood donation practice (Hosmer-Lemeshow χ²=14.92, df=8, p=0.061; Nagelkerke R²=0.163) are now reported in the main text. Sensitivity analyses for knowledge and attitude were already included in the supplementary table note.

**Reviewer #2**

**Comment 1: Questionnaire Development and Validation**

**Response:** No formal quantitative content validation (e.g., CVI) was conducted. However, the questionnaire was qualitatively validated through extensive literature review, peer review, and consultation with 2 public health faculty experts, plus pretesting with revisions. Construct validity (EFA S1, item-total S1), reliability reporting, and low α=0.43 implications (ceiling effect S4 Table) are comprehensively addressed in Reviewer 1 responses (Major Comments 3 and 7; Limitations).

**Comment 2: Sampling Strategy and Response Bias**

**Response:** This is comprehensively addressed in Reviewer 1, Major Comment 1 (Queries 1-2). Key points: (1) No respondent-nonrespondent demographic comparison possible (email-only access, no linked demographics); (2) 33.2% response rate discussed with female over-representation (26.7% vs. national engineering ~17%), indicating response-propensity bias; (3) Census-like invitation to all 576 students minimized access bias; (4) Limitations explicitly noted for gender generalizability.

**Comment 3: Sample Size Calculation and Study Power**

**Response:** This is comprehensively addressed in Reviewer 1, Major Comment 1 (Query 3). Key points: (1) Achieved n=191 exceeds unadjusted target n=176; (2) Post-hoc G*Power confirms 91% power for logistic regression (OR=2.0, n=191, α=0.05, EPV=8); (3) Adjusted target n=451 not reached due to 33.2% response rate (addressed in Queries 1-2); (4) Model fit confirmed acceptable (Hosmer-Lemeshow p=0.061, Nagelkerke R²=0.163) with all details now reported in manuscript.

**Comment 4: Statistical Analysis and Model Interpretation**

**Low R²=0.04 and Borderline p=0.060 Interpretation**

**Response:** This is already covered in Reviewer 1, Major Comment 4. We've addressed the low R²=0.04 and borderline p=0.060 with more cautious interpretation; explained continuous vs. dichotomized analyses in Methods; justified p≤0.20 screening with a prior study citation; and reported multicollinearity result (max VIF<2). All details are now in the manuscript.

**Comment 5: Interpretation of Gender Differences**

**Response:** Following your suggestion, we've now incorporated cultural factors (misconceptions, lack of personal request) and institutional factors (heavy engineering workload, lack of blood donation camps) into the Discussion alongside physiological hypotheses. All explanations appropriately use "may be attributed to" language and integrate both study findings and cited South Asia literature.

**Manuscript changes:** This paradox—where higher knowledge in females does not translate to practice—may be attributed to physiological barriers such as lower body weight, higher prevalence of anemia, or fear of needles, institutional factor such as heavy engineering workload, lack of organization of blood donation camps, and cultural factors such as misconception and lack of personal request made for the blood donation which are frequently reported obstacles for female donors in South Asia and in the current study (page 28, lines 525-530, tracked changes).

**Comment 6: Overly Lengthy Introduction and Discussion**

**Response:** We'd already condensed the Introduction's description in Reviewer 1 Minor Comment 1. For the Discussion, we've tightened the first paragraph in tracked changes by removing redundant summary.
